# Supplementary material for: Mechanism of N-Acetyl-D-alloisoleucine in Controlling Strawberry Black Root Rot
Source: Plants (Basel). 2025 Mar 6;14(5):829. doi: 10.3390/plants14050829 (PMC11902167; doi:10.3390/plants14050829)
Supplement: Supplementary file 1 [file plants-14-00829-s001.zip › supplementary materials/Table S1 (2)(1).docx]

**Table S1.** **Differential metabolites in *Bacillus subtilis* S-16**

| **Serial  number** | **Compound** | **Classification** | **Precursor type** | **Mass-to-charge ratio**  **（m/z）** | **Retention time (min)** | **Molecular  formula** |
| --- | --- | --- | --- | --- | --- | --- |
| 1 | Oxoadipic Acid | Medium-chain keto acids and derivatives | [M-H]- | 159.03017 | 2.411 | C_6_ H_8_O_5_ |
| 2 | 2-(3,4-dimethoxyphenyl)ethanamine | Methoxybenzenes | [M-H]- | 180.10338 | 5.504 | C_10_ H_15_NO_2_ |
| 3 | 4-Hydroxyphenylpyruvic acid | Phenylpyruvic acid derivatives | [M-H]- | 179.03539 | 5.711 | C_9_H_8_O_4_ |
| 4 | Pantetheine | Amino acids, peptides, and analogues | [M-H]- | 277.12259 | 5.488 | C_11_H_22_N_2_O_4_S |
| 5 | 5-Methyl-2'-deoxycytidine | Pyrimidine 2'-deoxyribonucleosides | [M-H]- | 240.09894 | 3.674 | C_10_H_15_N_3_O_4_ |
| 6 | 4-Hydroxybenzoic acid | Benzoic acids and derivatives | [M-H]- | 155.03529 | 6.006 | C_7_H_6_O_3_ |
| 7 | 4-Hydroxy-3-methoxymandelic acid | Anisoles | [M-H]- | 197.04568 | 4.908 | C_9_H_10_O_5_ |
| 8 | Sucrose | Carbohydrates and carbohydrate conjugates | [M-H]- | 341.10883 | 1.58 | C_12_H_22_O_11_ |
| 9 | 2-Hydroxycaproic acid | Fatty acids and conjugates | [M-H]- | 131.07149 | 5.678 | C_6_H_12_O_3_ |
| 10 | 3-Methyladipic acid | Fatty acids and conjugates | [M-H]- | 159.06671 | 5.475 | C_7_H_12_O_4_ |
| 11 | Indole-3-butyric acid | Indoles | [M-H]- | 202.08789 | 5.037 | C_12_H_13_NO_2_ |
| 12 | 2,5-Dihydroxybenzaldehyde | Carbonyl compounds | [M-H]- | 137.0246 | 5.295 | C_7_H_6_O_3_ |
| 13 | Adenosine diphosphate ribose | Purine nucleotide sugars | [M-H]- | 558.06445 | 1.613 | C_15_H_23_N_5_O_14_P_2_ |
| 14 | 2-Hydroxy-4-methylthiobutanoic acid | Fatty acids and conjugates | [M-H]- | 149.02805 | 5.268 | C_5_H_10_O_3_S |
| 15 | Fludrocortisone acetate | Pregnane steroids | [M-H]- | 421.20831 | 6.034 | C_23_H_31_FO_6_ |
| 16 | L-Asparagine | Amino acids, peptides, and analogues | [M-H]- | 131.04637 | 1.583 | C_4_H_8_N_2_O_3_ |
| 17 | 2-Hydroxyvaleric acid | Fatty acids and conjugates | [M-H]- | 117.05578 | 5.316 | C_5_H_10_O_3_ |
| 18 | 3-Indoleacrylic acid | Indoles | [M-H]- | 186.05635 | 5.621 | C_11_H_9_NO_2_ |
| 19 | Cytidine 5'-Monophosphate-N-Acetylneuraminic Acid | Pyrimidine nucleotide sugars | [M-H]- | 651.09808 | 1.982 | C_20_H_31_N_4_O_16_P |
| 20 | L-cysteine | Amino acids, peptides, and analogues | [M-H]- | 120.01277 | 4.945 | C_3_H_7_NO_2_S |
| 21 | 2-Aminobenzenesulfonic acid | Benzenesulfonic acids and derivatives | [M-H]- | 172.00786 | 4.93 | C_6_H_7_NO_3_S |
| 22 | 2-Oxobutyric acid | Short-chain keto acids and derivatives | [M-H]- | 101.02457 | 1.594 | C_4_H_6_O_3_ |
| 23 | Abametapir | Bipyridines and oligopyridines | [M+H] + | 185.10738 | 5.065 | C_12_H_12_N_2_ |
| 24 | 2-Methylpentanedioic acid | Fatty acids and conjugates | [M+H] + | 293.12238 | 5.548 | C_6_H_10_O_4_ |
| 25 | L-Glutamic acid | Amino acids, peptides, and analogues | [M+H] + | 148.06029 | 1.554 | C_5_H_9_NO_4_ |
| 26 | Oleanolic acid | Triterpenoids | [M+H] + | 457.3635 | 6.607 | C_30_H_48_O_3_ |
| 27 | Hypoxanthine | Purines and purine derivatives | [M+H] + | 137.04602 | 2.051 | C_5_H_4_N_4_O |
| 28 | 2,6-Dihydroxypurine | Purines and purine derivatives | [M+H] + | 153.04071 | 2.205 | C_5_H_4_N_4_O_2_ |
| 29 | Isophorone | Carbonyl compounds | [M+H] + | 139.11186 | 5.918 | C_9_H_14_O |
| 30 | D-Phenylalanine | Amino acids, peptides, and analogues | [M+H] + | 166.08685 | 12.05 | C_9_H_11_NO_2_ |
| 31 | 4'-(Imidazol-1-yl)acetophenone | Carbonyl compounds | [M+H] + | 187.08644 | 5.729 | C_11_H_10_N_2_O |
| 32 | L-arginine | Amino acids, peptides, and analogues | [M+H] + | 175.11888 | 5.086 | C_6_H_14_N_4_O_2_ |
| 33 | Choline | Quaternary ammonium salts | [M+H] + | 104.10696 | 1.435 | C_5_H_13_NO |
| 34 | Tetrahydrocortisone | Hydroxysteroids | [M+H] + | 365.22922 | 6.292 | C_21_H_32_O_5_ |
| 35 | Spermine | Amines | [M+H] + | 203.22266 | 1.237 | C_10_H_26_N_4_ |
| 36 | Indole-3-carboxaldehyde | Indoles | [M+H] + | 146.06039 | 5.371 | C_9_H_7_NO |
| 37 | Muscone | Carbonyl compounds | [M+H] + | 239.23633 | 6.688 | C_16_H_30_O |
| 38 | 2-Oxindole | Hydroxyindoles | [M+H] + | 134.0601 | 2.577 | C_8_H_7_NO |
| 39 | N-Acetyl-L-glutamate | Amino acids, peptides, and analogues | [M+H] + | 190.07031 | 1.525 | C_7_H_11_NO_5_ |
| 40 | Perillartine | Monoterpenoids | [M+H] + | 166.12277 | 6.447 | C_10_H_15_NO |
| 41 | 2-Hydroxybutyric acid | Alpha hydroxy acids and derivatives | [M+H] + | 105.05493 | 6.834 | C_4_H_8_O_3_ |
| 42 | Pyroglutamic acid | Amino acids, peptides, and analogues | [M+H] + | 130.04987 | 1.561 | C_5_H_7_NO_3_ |
| 43 | 2-Methoxyresorcinol | Methoxyphenols | [M+H] + | 159.06523 | 5.641 | C_7_H_8_O_3_ |
| 44 | 1-Palmitoylglycerol | Monoradylglycerols | [M+H] + | 331.28378 | 9.696 | C_19_H_38_O_4_ |
| 45 | N-Acetylneuraminic acid | Carbohydrates and carbohydrate conjugates | [M+H] + | 310.11243 | 1.543 | C_11_H_19_NO_9_ |
| 46 | Anserine | Hybrid peptides | [M+H] + | 241.1286 | 2.43 | C_10_H_16_N_4_O_3_ |
| 47 | D-Pantethine | Amino acids, peptides, and analogues | [M+H] + | 555.25189 | 5.553 | C_22_H_42_N_4_O_8_S_2_ |
| 48 | Bilirubin | Bilirubins | [M+H] + | 585.2702 | 4.845 | C_33_H_36_N_4_O_6_ |
| 49 | L-Pyroglutamic acid | Amino acids, peptides, and analogues | [M+H] + | 130.04987 | 2.21 | C_5_H_7_NO_3_ |
| 50 | Glycerol 1-hexadecanoate | Monoradylglycerols | [M+H] + | 331.284 | 7.724 | C_19_H_38_O_4_ |
| 51 | Asp-Phe | Amino acids, peptides, and analogues | [M+H] + | 281.11374 | 5.355 | C_13_H_16_N_2_O_5_ |
| 52 | Glutathione | Amino acids, peptides, and analogues | [M+H] + | 308.09076 | 5.683 | C_10_H_17_N_3_O_6_S |
| 53 | Kahweol | Monoterpenoids | [M+H] + | 315.19516 | 6.621 | C_20_H_26_O_3_ |
| 54 | Styrene | Styrenes | [M+H] + | 105.0699 | 5.228 | C_8_H_8_ |
| 55 | N-Acetylornithine | Amino acids, peptides, and analogues | [M+H] + | 175.1081 | 1.52 | C_7_H_14_N_2_O_3_ |
| 56 | L-Glutamate | Amino acids, peptides, and analogues | [M+H] + | 148.06035 | 2.517 | C_5_H_9_NO_4_ |
| 57 | 1,3-Dimethyluracil | Pyrimidines and pyrimidine derivatives | [M+H] + | 141.06596 | 2.312 | C_6_H_8_N_2_O_2_ |
| 58 | D-Erythrose 4-phosphate | Carbohydrates and carbohydrate conjugates | [M+H] + | 201.01634 | 4.841 | C_4_H_9_O_7_P |
| 59 | L-Ornithine | Amino acids, peptides, and analogues | [M+H] + | 133.09769 | 1.217 | C_5_H_12_N_2_O_2_ |
| 60 | 2-Deoxyuridine | Pyrimidine 2'-deoxyribonucleosides | [M+H] + | 229.08133 | 1.536 | C_9_H_12_N_2_O_5_ |
| 61 | N6-Acetyl-L-lysine | Amino acids, peptides, and analogues | [M+H] + | 207.13327 | 1.217 | C_8_H_16_N_2_O_3_ |
| 62 | Quercetin | Flavones | [M+H] + | 321.05566 | 8.96 | C_15_H_10_O_7_ |
| 63 | 2-Aminopimelic acid | Amino acids, peptides, and analogues | [M+H] + | 176.09134 | 1.57 | C_7_H_13_NO_4_ |
| 64 | Creatine | Amino acids, peptides, and analogues | [M+H] + | 301.10156 | 2.81 | C_4_H_9_N_3_O_2_ |
| 65 | Tryptophanol | Indoles | [M+H] + | 191.11743 | 5.197 | C_11_H_14_N_2_O |
| 66 | DL-Panthenol | Fatty amides | [M+H] + | 206.13853 | 3.82 | C_9_H_19_NO_4_ |
| 67 | Andrographolide | Gamma butyrolactones | [M+H] + | 373.19876 | 6.632 | C_20_H_30_O_5_ |
| 68 | N-Methylanthranilic acid | Benzoic acids and derivatives | [M+H] + | 152.07077 | 2.634 | C_8_H_9_NO_2_ |
|  |  |  |  |  |  |  |
|  |  |  |  |  |  |  |
