# Supplementary material for: Mechanism of N-Acetyl-D-alloisoleucine in Controlling Strawberry Black Root Rot
Source: Plants (Basel). 2025 Mar 6;14(5):829. doi: 10.3390/plants14050829 (PMC11902167; doi:10.3390/plants14050829)
Supplement: Supplementary file 1 [file plants-14-00829-s001.zip › supplementary materials/Table S2.docx]

**Table S2.** **Comparison of differences in *Bacillus subtilis* S-16 fermentation broth**

**vs control metabolites**

| **Types** | **S-16 vs Control** | | |
| --- | --- | --- | --- |
|  | Total | Up-regulation  quantity | Down-regulation  quantity |
| Amino acids, peptides,  and analogues | 18 | 3 | 15 |
| Carbohydrates and  carbohydrate conjugates | 3 | 0 | 3 |
| Carbonyl compounds | 4 | 2 | 2 |
| Fatty acids and  conjugates | 5 | 3 | 2 |
| Indoles | 4 | 0 | 4 |
| Monoterpenoids | 2 | 1 | 1 |
| Purines and purine  derivatives | 2 | 1 | 1 |
| Others | 30 | 7 | 23 |
| Total | 68 | 17 | 51 |
