# Supplementary material for: Mechanism of N-Acetyl-D-alloisoleucine in Controlling Strawberry Black Root Rot
Source: Plants (Basel). 2025 Mar 6;14(5):829. doi: 10.3390/plants14050829 (PMC11902167; doi:10.3390/plants14050829)
Supplement: Supplementary file 1 [file plants-14-00829-s001.zip › supplementary materials/Table S3 (2).docx]

**Table S3. Primers used in the study**

| Primers | Sequence shown in 5’→3’ orientation |
| --- | --- |
| EF1-a-F | CCTCAAGACATTCTCAGTAAAG |
| EF1-a-R | CTCCGGATATGTTGCGTCAA |
| PR1-F | TACTCCATTCCACTCATGCACAA |
| PR1-R | ATGCACGAGGTTGCAGTCGCCAT |
| PAL-F | GTGAAAGAAGCGAAGAAGG |
| PALR | GAAGCTCGGAGCAGTATG |
| NPR1-F | ATCAGAAGCAACTTTGGAAGGTAGA |
| NPR1-R | ACCGCCATAGTGGCTTGTTT |
| PR5-F | CGTAGTTAGGTCCACCGAAGCATGTA |
| PR5-R | ACCTCCTAATGACACTCCCGAAACA |
| PDF1-F | ACCTCCTAATGACACTCCCGAAACA |
| PDF1-R | ATGGCTTGAAGCACATGCATTTTC |
| Fa-β-actin-F | ACCCAACCCCATTCTATTCT |
| Fa-β-actin-R | ATCATCTCCAGCAAACCCT |
