# Supplementary figures and images for: Mechanism of N-Acetyl-D-alloisoleucine in Controlling Strawberry Black Root Rot
Source: Plants (Basel). 2025 Mar 6;14(5):829. doi: 10.3390/plants14050829 (PMC11902167; doi:10.3390/plants14050829)

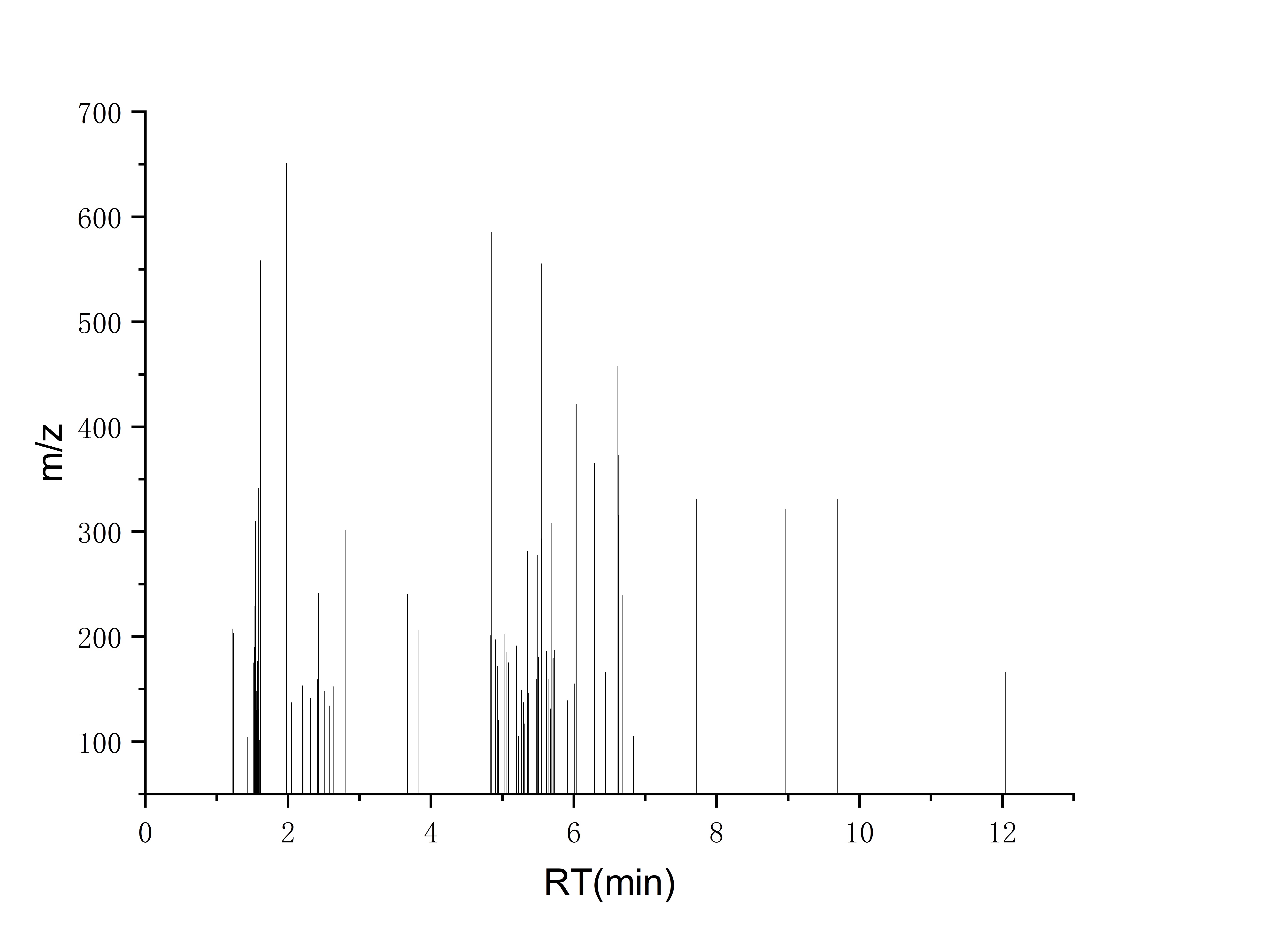

Supplement: Supplementary file 1 [file plants-14-00829-s001.zip › supplementary materials/Figure S1.png]
